# Mechanistic study on the restoration of intestinal barrier integrity and alleviation of inflammatory bowel disease by rhynchophylline via the AhR-NR4A1 pathway

**DOI:** 10.3389/fphar.2025.1629012

**Published:** 2025-09-15

**Authors:** Xuwen Mao, Lufeng Cheng, Yu Liu, Yuche Wu

**Affiliations:** ^1^ College of Pharmacy, Xinjiang Key Laboratory of Biopharmaceuticals and Medical Devices, Xinjiang Medical University, Urumqi, China; ^2^ Xinjiang Technical Institute of Physics and Chemistry, Chinese Academy of Sciences, Urumqi, China

**Keywords:** rhynchophylline, inflammatory bowel diseases, intestinal barrier integrity, AhR, Nr4a1

## Abstract

**Background:**

Restoring intestinal barrier function is considered an effective strategy for the treatment of Inflammatory Bowel Disease (IBD). Rhynchophylline (Rhy), a bioactive alkaloid sourced from the traditional herbs Uncariarhynchophylla used in Chinese medicine, is known for its antihypertensive, anti-asthmatic, and antitumor properties. This study explores the pharmacological effects and molecular mechanisms of Rhy in treating IBD.

**Methods:**

In vitro cell inflammation injury model was established, and the inflammatory factors, cell permeability, cell proliferation, and intercellular tight junction protein expression were measured after Rhy intervention, which verified the anti-inflammatory activity and enhancement of intestinal barrier function of Rhy. In vivo animal model of acute and chronic colitis was established, and Rhy was administered orally at three dose levels to evaluate the protective effects of Rhy on acute and chronic colitis in animals. The signaling pathways that enhance the intestinal barrier function of Rhy were identified through transcriptomics, gene knockout techniques, and molecular dynamics simulations.

**Results:**

In vitro results indicate that the levels of inflammatory markers–including IL-6, TNF-α, and NO–can be decreased by Rhy, demonstrating anti-inflammatory activity. It significantly lowers cellular permeability, promotes the proliferation of intestinal epithelial cells, and upregulates the expression of tight junction proteins Claudin4, Occludin, and ZO-1. These effects improve cellular morphology and improve the robustness of the intestinal barrier. In vivo, using three types of colitis animal models, Rhy shows significant protective effects on mice with acute and chronic colitis. It markedly reduces weight loss and Disease Activity Index (DAI) scores, prevents colonic shortening, reduces intestinal permeability, and decreases serum levels of IL-6, TNF-α, CXCL1, and IL-1β. These effects restore the structural integrity of colonic tissues and alleviate symptoms of acute and chronic colitis. Additionally, Rhy activates the AhR-NR4A1 pathway. This activation upregulates Claudin4, Occludin, and ZO-1 to repair the intestinal barrier and exert anti-colitic effects.

**Conclusion:**

These findings highlight Rhy improve the function and inhibits inflammation, providing dual beneficial activities in the epithelial lining of the gut to prevent and treat colitis.

## Graphical Abstract

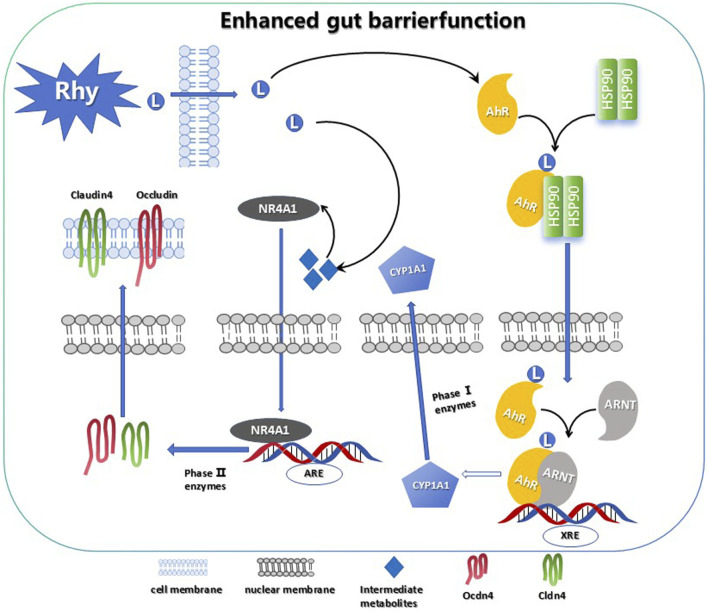

## 1 Introduction

Inflammatory Bowel Diseases (IBD) comprises idiopathic, recurring conditions marked by the disruption of the intestinal mucosal barrier, extensive infiltration of inflammatory cells, and abnormal activation of the immune system. Although the exact causes of IBD are not fully understood, these factors are considered critical in driving the progression of IBD (Cantón et al., 2024; Levy et al., 2017). Impairment of the intestinal barrier function can lead to dysbiosis of the gut microbiome, allowing various harmful substances—such as pathogens, endotoxins, and allergens—to cross the intestinal barrier into the bloodstream. This results in excessive immune responses and inflammatory reactions (Sonnenburg and Bäckhed, 2016; Belkaid and Hand, 2014). Tight junctions, positioned at the apex of intestinal epithelial cells, are essential for maintaining the integrity of the intestinal barrier. In pathological conditions, these tight junctions experience compromised structure and function, exacerbating disease progression in patients with IBD (Quaglio et al., 2022; Gop et al., 2018). Therefore, restoring the integrity of tight junctions could be an effective treatment approach for IBD.

Gouteng (Uncaria rhynchophylla), a traditional Chinese herb from the Rubiaceae family, recognized for its hooked stems used in medicine. It clears heat (reduces inflammation), calms the liver (alleviates hypertension and dizziness), and stops internal wind (controls tremors and seizures). Clinically applied for high blood pressure, pediatric fever-induced convulsions, and anxiety disorders. Modern research attributes these effects to alkaloids like rhynchophylline (Yang et al., 2020; Chen et al., 2024). This plant contains various bioactive alkaloids, among which Rhynchophylline (Rhy) is one of the primary active components. Rhy is noted for its effects in lowering blood pressure, alleviating asthma, and exhibiting anti-tumor properties, showing significant potential for further development (Ballester Roig et al., 2021; Jiang et al., 2023; Wang et al., 2023). Studies have demonstrated that Rhy can reduce generation of pro-inflammatory mediators, including TNF-α and IL-1β, and exhibits anti-inflammatory and antioxidant characteristics by inhibiting the TLR4/NF-κB/NLRP3 signaling pathway (Geetha and Ramachandran, 2021). Currently, the therapeutic effects and targets of Rhy in the treatment of IBD remain unclear.

We evaluated Rhy’s activity in this research and discovered that, in addition to its anti-inflammatory properties, it also enhances intestinal barrier function. We demonstrated that Rhy activates the aryl hydrocarbon receptor (AhR)-nuclear receptor subfamily four group A member 1 (NR4A1) pathway, inducing increased expression of tight junction proteins Claudin4, Occludin, and ZO-1, thereby reducing intestinal permeability and enhancing barrier function. Furthermore, oral administration of Rhy significantly alleviated systemic inflammation and symptoms of colitis, suggesting its potential therapeutic role in IBD and indicating that Rhy might offer a promising treatment option for IBD.

## 2 Methods

### 2.1 Materials

Rhynchophylline (Rhy) was acquired from Shanghai Yuanye Bio-Technology Co., Ltd. (batch number C22M11S113789). Dextran Sodium Sulfate (DSS) was purchased from Zhejiang Hisun Pharmaceutical Co., Ltd. (batch number C12750877). Mouse fecal occult blood test kits were obtained from Shanghai Enzyme-linked Biotechnology Co., Ltd. (batch number 0701A22). ELISA kits were sourced from Shanghai Jianglai Biotechnology Co., Ltd., and all antibodies were purchased from Beijing Biosynthesis Biotechnology Co., Ltd. Mouse monocyte-macrophage cells (RAW264.7) and human colorectal cancer cells (Caco2) were acquired from Wuhan Punuosai Life Science Co., Ltd. (product numbers CL-0190 and CL-0050), and human colon epithelial cells (NCM460) were purchased from Shanghai Jining Industrial Co., Ltd. (product number JN26629).

### 2.2 Experimental animals

Sixty 5-week-old male C57BL/6J mice, weighing between 20 ± 2 g, were procured from Beijing Vital River Laboratory Animal Technology Co., Ltd. (license number SCXK (Jing) 2016–0006). These mice were maintained at the Laboratory Animal Center of Xinjiang Medical University, which operates under specific pathogen-free (SPF) conditions, with license SYXK (Xin) 2016–0002. The housing conditions were maintained with a 12 h light/12 h dark cycle, at a temperature of 21 °C ± 2 °C, and a humidity of 40%–45%. The experiment was approved by the Ethics Committee of the Xinjiang Medical University Laboratory Animal Center, ethical approval number: IACUC-20211016–39. All mice were acclimated for 1 week before the experiments. Mice were euthanized by intraperitoneal injection of 3% sodium pentobarbital solution.

### 2.3 Cell culture and treatment

Cells were cultured in DMEM enriched with 10% fetal bovine serum and 1% antibiotics (penicillin 100 U/ml, streptomycin 100 μg/mL) at 37 °C in an atmosphere of 5% CO_2_. To examine the anti-inflammatory properties of Rhy, RAW264.7 cells were first exposed to 50 ng/mL LPS (derived from *Escherichia coli* O55:B5, Sigma, L2880) for 6 h to induce immune-inflammatory damage (METTL3, 2024), followed by treatment with varying concentrations of Rhy (0.5, 1, 5, 10, and 50 μM) for another 6 h. The experiment was conducted four times, measuring levels of IL-6, TNF-α, and NO in the supernatants of RAW264.7 cell cultures using ELISA.

### 2.4 Cell proliferation of NCM460 was determined by CCK-8 assay

Additionally, to evaluate the effect of Rhy on the viability of NCM460 cells, these cells were co-cultured with 2% DSS for 24 h to mimic chemical-induced inflammation (Chassaing et al., 2014), then treated with Rhy at concentrations of 0.05, 0.5, 5, 50, and 100 μM for 24 h. CCK-8 solution was then added, and the mixture was gently shaken and incubated for another 4 h. The absorbance (OD value) was detected at a wavelength of 450 nm using a microplate reader. The experiment was repeated five times to assess potential impacts on cell viability.

### 2.5 DSS-induced colitis

Acute colitis was induced in C57BL/6J mice using DSS. We randomly divided the mice into these groups: Control, Model, Dexamethasone (DXM, 4 mg kg^-1^), and Rhy low (Rhy-L, 2 mg kg^-1^), medium (Rhy-M, 4 mg kg^-1^), and high dose (Rhy-H, 8 mg kg^-1^) groups. The control group mice were provided with regular food and drinking water daily. They were not subjected to any treatment. The model, DXM, and Rhy (low, medium, high dose) dose groups were given 3% DSS solution in place of drinking water, administered *ad libitum*, and were allowed free access to food. Mice in the Rhy (low, medium, high dose) groups were orally administered Rhy solution (2 mg kg^-1^, 4 mg kg^-1^, 8 mg kg^-1^) once per day for 7 days in a row. For the chronic DSS colitis model, a 2% DSS regimen was used over four cycles, with each DSS cycle lasting 7 days, followed by a 14-day rest period during which mice drank regular water, and Rhy (4 mg kg^-1^) was administered for 7 days during each DSS cycle.

### 2.6 Assessment of colitis severity and tissue collection

The mice were assessed daily for body weight, fecal consistency, and rectal bleeding to compute the Disease Activity Index (DAI) (Mulberry Anthocyanins Ameliorate, 2024). Following euthanasia, the colon was excised and washed with PBS containing 1 mM Phenylmethanesulfonyl fluoride (PMSF) and 0.2 mM sodium orthovanadate. Measurements of colon length and weight were taken, and sections of the colon were sampled for myeloperoxidase (MPO) activity tests and RNA extraction. Tissues designated for MPO analysis and RNA extraction were quickly frozen in liquid nitrogen and stored at −80 °C for subsequent analysis. Samples for histological evaluation were preserved in a solution containing 10% neutral buffered formalin. Blood samples were collected, centrifuged at 3,500 rpm for 15 min to separate serum, and serum levels of cytokines (IL-6, TNF-α, IL-1β) and chemokines (CXCL1) were determined using ELISA kits.

### 2.7 Lipopolysaccharide-induced peritonitis

Male C57BL/6J mice, aged 6–8 weeks, were randomly assigned into three groups: Control, Model, and Rhy group. The control and model groups received the same volume of 0.25% sodium carboxymethyl cellulose (CMC-Na) orally, while the Rhy group was administered 4 mg/kg Rhy via gavage at 0, 6, 12, 18, and 24 h. After 24 h, peritonitis was induced in the mice by intraperitoneal injection of LPS (2 mg kg^-1^). The mice were euthanized 6 h later, and blood samples were collected for analysis of IL-6, TNF-α, and NO using ELISA kits.

### 2.8 RT-PCR

Total RNA was isolated from cells/tissues using an RNA tissue kit, and reverse transcription was performed using the TaqMan Reverse Transcription kit. The synthesized cDNA was then mixed with 100 nM real-time PCR primers LLC and 1× TB Green Premix Ex Taq II for gene expression analysis using the CFX96™ Real-Time System (Bio-Rad). Gene expression changes were calculated using the 2^−ΔΔCT^ method with GAPDH/β-actin as internal reference genes, normalized to untreated controls.

### 2.9 Intestinal permeability assessment

Intestinal permeability was evaluated both *in vitro* and *in vivo*. *In vitro*, Caco-2 cells were grown on transwell plates for 19–21 days until resistance values stabilized, indicating a formed cellular barrier. The upper chamber received 100 µL of 0.1 mg/mL FITC-Dextran (molecular weight 4 kDa), and the lower chamber received 600 µL PBS. After 60 min of incubation in a CO_2_ incubator, 100 µL from the lower chamber was analyzed for fluorescence intensity on a 96-well plate.

*In vivo*, mice were fasted for 4 h prior to euthanasia and orally administered FITC-Dextran (60 mg/100 g body weight). The concentration of FITC-Dextran in the serum was then measured.

### 2.10 Phalloidin assay to assess the impact of rhynchophylline on the cytoskeleton of Caco-2 cells

Caco-2 cells were divided into Control, Model, and Rhynchophylline (50 μM) groups, and fixed with 4% formaldehyde for 10 min. The cells were rinsed 2–3 times with PBS, followed by permeabilization with 5 mg mL^-1^ permeabilization solution (PBS containing Triton X-100) for 5 min. The cells were incubated in the dark at room temperature with 200 μL of rhodamine-phalloidin (100 nM) for 30 min. After washing with PBS, the cells were stained with 100 nM DAPI (1 μg mL^-1^) for 5–10 min to label the nuclei. The cells were rinsed again with PBS and treated with an anti-fade reagent before fluorescence detection at a wavelength of 440 nm.

### 2.11 EdU (5-Ethynyl-2′-deoxyuridine) assay to evaluate the Effect of Rhynchophylline on Caco-2

Cell Proliferation Caco-2 cells were divided into Control, Model, and Rhynchophylline (50 μM) groups. Cells were adjusted to a density of 1 × 10^5^ cells per well and seeded in four-quadrant laser confocal dishes, with 500 μL per quadrant. Add a 2× EdU working solution, prepared by diluting 10 μM EdU in cell culture medium at a 1:500 ratio, to the cells and incubate for 2 h. Cells were fixed with 4% formaldehyde for 15 min. After fixation, they were washed with a 3% BSA washing solution. Permeabilization was carried out with 0.3% Triton X-100 in PBS for 10–15 min. After washing, a Click reaction was performed by adding 0.5 mL of Click reaction mixture per well (comprising Click Reaction Buffer, CuSO_4_, Azide 555, and Click Additive Solution, mixed in the proportions of 2.15 mL, 100 μL, 5 μL, and 250 μL respectively) and incubating in the dark at room temperature for 30 min. After another wash, cells were stained with 1 mL of 1× Hoechst solution per well and incubated in the dark at room temperature for 10 min before analysis.

### 2.12 Western blot to detect protein expression in colonic tissue

Colonic tissue samples were lysed using Lysis Buffer to extract proteins, which were then separated by centrifugation. The lysed protein samples were loaded onto polyacrylamide gels for electrophoresis. After electrophoresis, proteins on the polyacrylamide gel were transferred to a PVDF membrane. The membrane was blocked with 5% skim milk for 1 h and incubated with the primary antibody at 4 °C for 24 h, followed by washing. Subsequently, the membrane was incubated with a secondary antibody at room temperature for 1 hour and then washed again. The membrane’s protein expression was visualized with ECL and analyzed with ImageJ software. Concurrent with each target gene detection, the reference gene is simultaneously assayed in triplicate to ensure synchronous detection of both the reference and target genes, thereby maintaining the stability of the reference gene across all target gene detection cycles.

### 2.13 Transcriptomic analysis

Transcriptomic analysis was conducted by Biotree Technology Co., Ltd. (Shanghai, China). An mRNA sequencing library was prepared from 5 μg of total RNA, and its concentration was quantified using the Qubit fluorometric quantitation method. Sequencing was performed on the HiSeq X10 system (Illumina, San Diego, CA, USA). Colonic tissues from the control group (Group A), model group (Group B), and Rhy group (Group C) mice were collected. Total RNA was extracted from the colonic tissues of each experimental group using RNAiso Plus (TaKaRa, Japan), and the integrity of the isolated RNA was assessed using an Agilent 2,100 bioanalyzer (RIN >9.5).

### 2.14 Molecular docking of Rhy with AhR

The X-ray crystal structure of the AhR_HUMAN protein (Uniprot ID: P35869) was obtained from the Protein Data Bank (PDB ID: 5NJ8) with a resolution of 3.30 Å (Dong et al., 2021). The structure of Rhynchophylline (CAS: 76–66–4) was retrieved from the PubChem database. To refine the structure, PyMOL (Version 2.4, New York, NY, USA) was used to remove water and miscellaneous molecules (hydrogen atoms and charges) from the protein. The LCR molecule and Sirt protein family were formatted in pdbqt using AutoDockTools (Autodock 1.5.7, Scripps Research Institute, San Diego, USA), and molecular docking was performed using Autodock Vina (version 1.1.2) (Pinzi and Rastelli, 2019). The docking images were visualized using PyMOL.

### 2.15 Molecular dynamics simulation of Rhy with AhR

Molecular dynamics simulations were used to explore the spatial conformational changes of the Rhy-AhR complex. The root mean square deviation (RMSD) was calculated to assess the overall stability and variations of the molecular structure. The root mean square fluctuation (RMSF) measured the degree of oscillation for each atom during the simulation, revealing the flexibility and dynamic behavior of individual atoms. The radius of gyration (RG) was calculated to evaluate the protein’s shape and volume. The solvent accessible surface area (SASA) was calculated to assess the protein’s surface area available for interaction with the solvent, reflecting the extent to which the protein unfolds in solution. These metrics were used to evaluate the dynamic equilibrium of the Rhy-AhR system.

### 2.16 Immunofluorescence detection of intestinal barrier protein expression in Caco-2 cells

Caco-2 cells (5 × 10^4^ per well) were seeded into a 6-well plate and incubated at 37 °C in a CO_2_ incubator for 24 h. The cells were divided into Control group (0.01% DMSO) and Rhy group (50 µM). The cells were fixed with a 4% solution of formaldehyd. They were then stained with antibodies against Claudin4, Occludin, and ZO-1 diluted at 1:2000 ratio, followed by labeling with fluorescent tags. After primary antibodies were collected, cells were washed with PBS, followed by a 1-h incubation with secondary antibodies, and a subsequent rinse with PBS, and then stained with DAPI for 5 min (diluted at 1:100 ratio). Confocal microscopy was used to capture images of the labeled cells at last.

### 2.17 Cyp1A1 enzyme activity assay

Three groups were formed from the mice: Vehicle, Rhy, and FICZ (6-Formylindolo [3,2-b]carbazole, a potent AhR agonist) groups, receiving oral administration daily for 1 week. One week after the experiment began, mice were euthanized, and their colon and liver tissues were harvested to extract microsomes. Initially, the liver was rinsed with 0.9% sodium chloride solution to remove adhering blood and solution. The tissues were then homogenized in a buffer containing 50 mM Tris-HCl (pH 7.4) and 250 mM sucrose. The homogenate was centrifuged at 4 °C at 10,000 rpm for 30 min. Subsequently, the supernatant was further centrifuged at 105,000 rpm at 4 °C for 60 min. The resulting pellet was resuspended in the supernatant from the first centrifugation and was used for subsequent protein content and CYP enzyme activity analysis.

### 2.18 Ethoxyresorufin-O-deethylase (EROD) assay

0.5 mg of microsomal protein was mixed with 200 µL of Tris buffer (0.1 M, pH 7.4) containing 0.01 mM ethoxyresorufin. The reaction was initiated by adding 0.1 mM NADPH and incubated at 37 °C for 10 min. To stop the reaction, an equal volume of acetonitrile was added. The mixture was then centrifuged at 13,000 rpm at 4 °C for 10 min. The resorufin levels in the supernatant were determined by a fluorescence assay.

### 2.19 P450-Glo Cyp1A1 luminescence assay

NCM460 cells were seeded at 2.5 × 10^4^ cells per well in a 48-well plate and treated with various concentrations of Rhy (0.1, 1, 10, 25, 50 µM) and FICZ (0.1, 1, 10, 25, 50 nM) for 24 h. Post-treatment, the medium was replaced with fresh medium containing Cyp1A1 substrate and incubated further. Then, 25 µL of medium from each well was transferred to a white opaque 96-well plate, and 25 µL of luciferase detection reagent was added to trigger the luminescent reaction. The reaction was incubated at room temperature for 20 min before measuring the luminescence.

### 2.20 Gene knockdown experiments

AhR siRNA (abx907072) and NR4A1 siRNA (abx926351) were obtained from Amyjet Scientific. In the knockdown experiments, Caco-2 cells (0.5 × 10^6^ per well) were seeded in a 6-well plate and cultured in a 37 °C CO_2_ incubator for 24 h. According to the manufacturer’s instructions, the cells were transfected with AhR, NR4A1, and control siRNAs using Lipofectamine RNAiMAX reagent (ThermoFisher Scientific, CA, USA). After 24 h of transfection, cells were treated either with the negative control (0.01% DMSO) or 50 µM Rhy for another 24 h. Following treatment, cells were lysed with RIPA buffer, and the expression levels of AhR, NR4A1, Claudin4, Occludin, and ZO-1 proteins were analyzed using Western blotting.

### 2.21 Statistical analysis

All result analyses were realized with the application of GraphPad Prism version 9. For continuous variables, the presentation was in the form of mean ± standard deviation; categorical variables were reported as percentages. Experimental results between the two groups were analyzed using unpaired Student’s *t* tests. For the results among more than two groups, a one-way analysis of variance (ANOVA) was implemented, followed by Tukey’s *post hoc* test to compare all the groups. Two-way ANOVA with Bonferroni *post hoc* testing was applied to assess the effects of two independent factors on measured variables. Non-normally distributed data were analyzed using the Kruskal–Wallis test for multi-group comparisons. *P* < 0.05 is regarded as a significant difference. Figures were plotted with the assistance of GraphPad Prism version 9.

## 3 Results

### 3.1 Anti-inflammatory activity and induction of tight junction protein expression by Rhy

**FIGURE 1:**
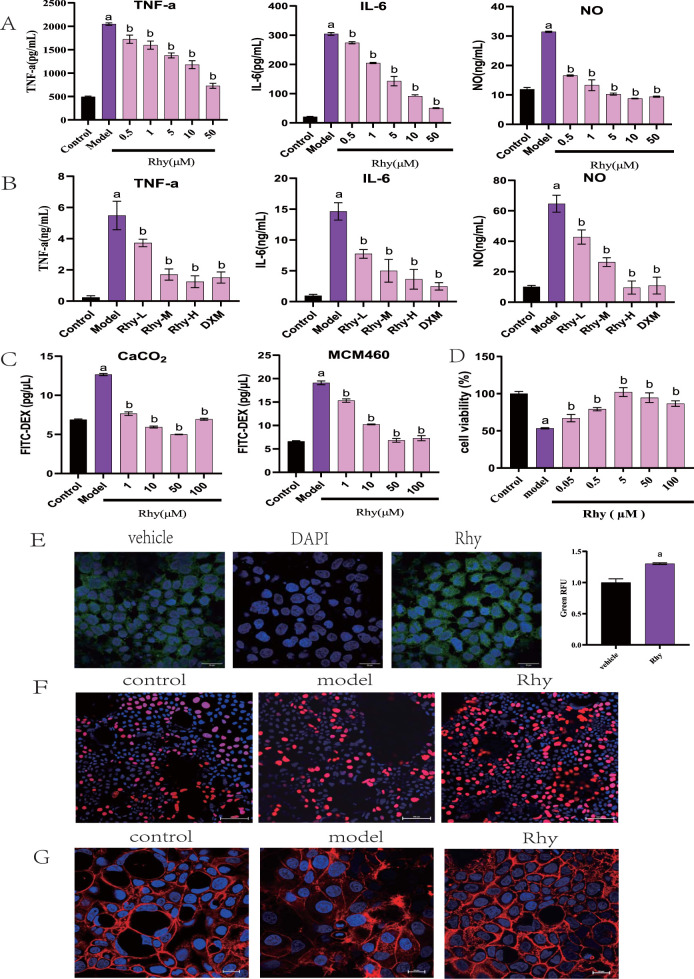
Rhy is a potent anti-inflammatory chemical compound and induces tight junction proteins. **(A)** RAW264.7 were stimulated with LPS without or with Rhy. IL-6, TNF-α and NO levels in supernatants were measured (n = 4). **(B)** C57BL/6j mice were pretreated with Rhy, serum levels of IL-6, TNF-α and NO was measured (n = 4). **(C)** FITC dextran was added to Caco2 or NCM460 cells to measure effect of Rhy on permeability of cells (n = 3). **(D)** CCK-8 method was used to detect the effect of Rhy on the proliferation of NCM460 cells (n = 5). **(E)** The level of Cldn4 protein was measured by immunofluorescence (n = 3). Scale bars for Caco2 cells indicate 50 μm respectively. **(F)** The effect of Rhy on the proliferation of Caco2 cells was detected by EDU method (n = 3). The cells were stained with 5-acetylene-2′-deoxyuridine. **(G)** Effect of Rhy on the cytoskeleton of Caco2 cells (n = 3). The cells were stained with rhodamine-phalloidin. ^a^ *P* < 0.05, indicating comparison with control group; ^b^ *P* < 0.05, indicating comparison with model group. Data were expressed as mean ± SEM.

Rhynchophylline (Rhy) significantly reduced the levels of IL-6, TNF-α, and NO in LPS-induced RAW264.7 mouse monocyte-macrophage cells, demonstrating anti-inflammatory activity at concentrations as low as 0.5 μM (Figure 1A). In a mouse model of LPS-induced peritonitis, treatment with Rhy significantly reduced serum levels of IL-6, TNF-α, and NO (Figure 1B). These results confirm Rhy’s anti-inflammatory properties. Rhy treatment significantly inhibited the increase in permeability induced by LPS in Caco-2 and NCM460 cells (Figure 1C). Concentrations of Rhy ranging from 0.05 to 100 µM promoted the proliferation of NCM460 cells, with the most pronounced effect observed at 5 µM (Figure 1D). Confocal imaging confirmed a significant increase in the expression of the tight junction protein Claudin4 in cells treated with Rhy (Figure 1E). Rhy also effectively reversed the inhibitory effect on cell proliferation induced by DSS in Caco-2 cells (Figure 1F) and improved the fibrotic changes and irregularities in cell morphology caused by DSS, restoring cells to a normal state (Figure 1G). These results indicate that Rhy can enhance the expression of tight junction proteins, improve intestinal cell morphology, and strengthen the integrity of the intestinal barrier.

### 3.2 Rhy alleviates acute colitis in mice

**FIGURE 2:**
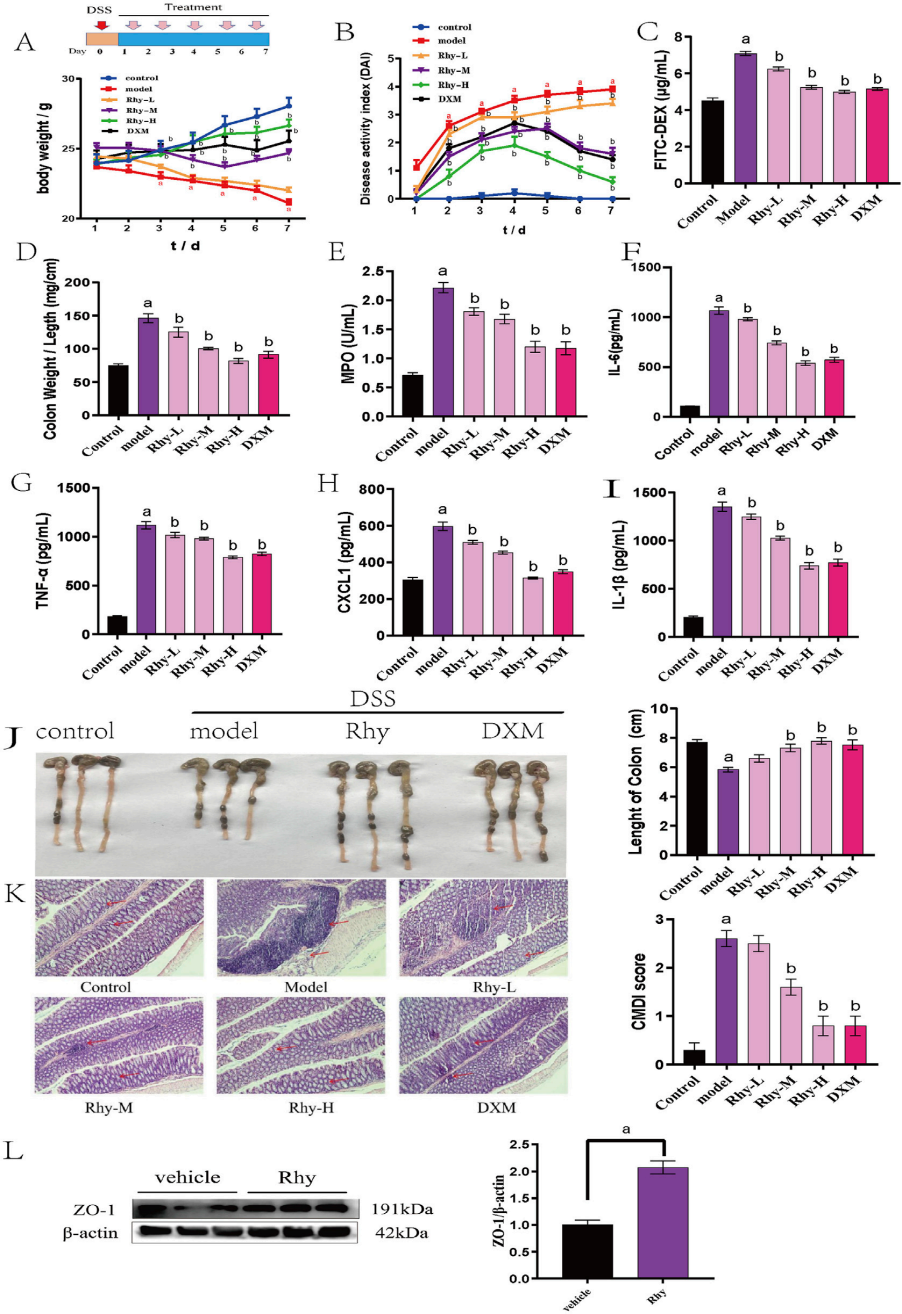
Rhy treatment attenuates DSS-induced colitis in mice (n = 10). **(A)** Body weight loss. **(B)** Disease activity index. **(C)** Intestinal permeability. **(D)** Ratio of colon weight/length. **(E)** Effect of Rhy on the levels of inflammatory factors MPO in the colon homogenate of IBD mice. **(F)** IL-6 level. **(G)** TNF-α level. **(H)** CXCL-1 level. **(I)** IL-1β level. **(J)** Colon lengths. **(K)** Microphotographs of hematoxylin and eosin (H&E) stained sections of colons and inflammation scores are shown. Scale bar indicates 100 μm. **(L)** ZO-1 expression in the colons of these mice was measured by immunoblots and quantified (n = 3). ^a^ *P* < 0.05, indicating comparison with control group, ^b^ *P* < 0.05, indicating comparison with model group. Data were expressed as mean ± SEM.

Rhy provided significant protection against 3% DSS-induced acute colitis in mice. The model group’s Disease Activity Index (DAI) scores increased progressively, with visible bloody stool occurring between days 5 and 7, indicating successful model induction (P < 0.01). Compared to the model group, Rhy significantly mitigated weight loss and DAI scores in colitis mice, prevented colonic shortening, and reduced intestinal permeability, suggesting an alleviation of colitis symptoms (Figures 2A–D,J). Rhy reduced myeloperoxidase (MPO) activity (Figure 2E), indicating a decrease in neutrophil infiltration. Additionally, Rhy lowered serum levels of inflammatory cytokines IL-6, TNF-α, CXCL1, and IL-1β in colitis mice (Figures 2F–I).

HE staining of colonic tissues from IBD model mice showed severe mucosal erosion, evident epithelial defects, and destruction of crypt architecture, with extensive infiltration of inflammatory cells in the mucosa and submucosa. After treatment with Rhy, the structural integrity of colonic tissues was largely restored, with reduced inflammatory cell infiltration. Additionally, goblet cells were neatly arranged, and colonic inflammation was significantly improved (Figure 2K). Furthermore, Western blot analysis confirmed that Rhy significantly upregulated ZO-1 protein levels, indicating its protective capacity for the intestinal barrier in mice (Figure 2L).

### 3.3 Rhy treatment alleviates chronic colitis in mice

**FIGURE 3:**
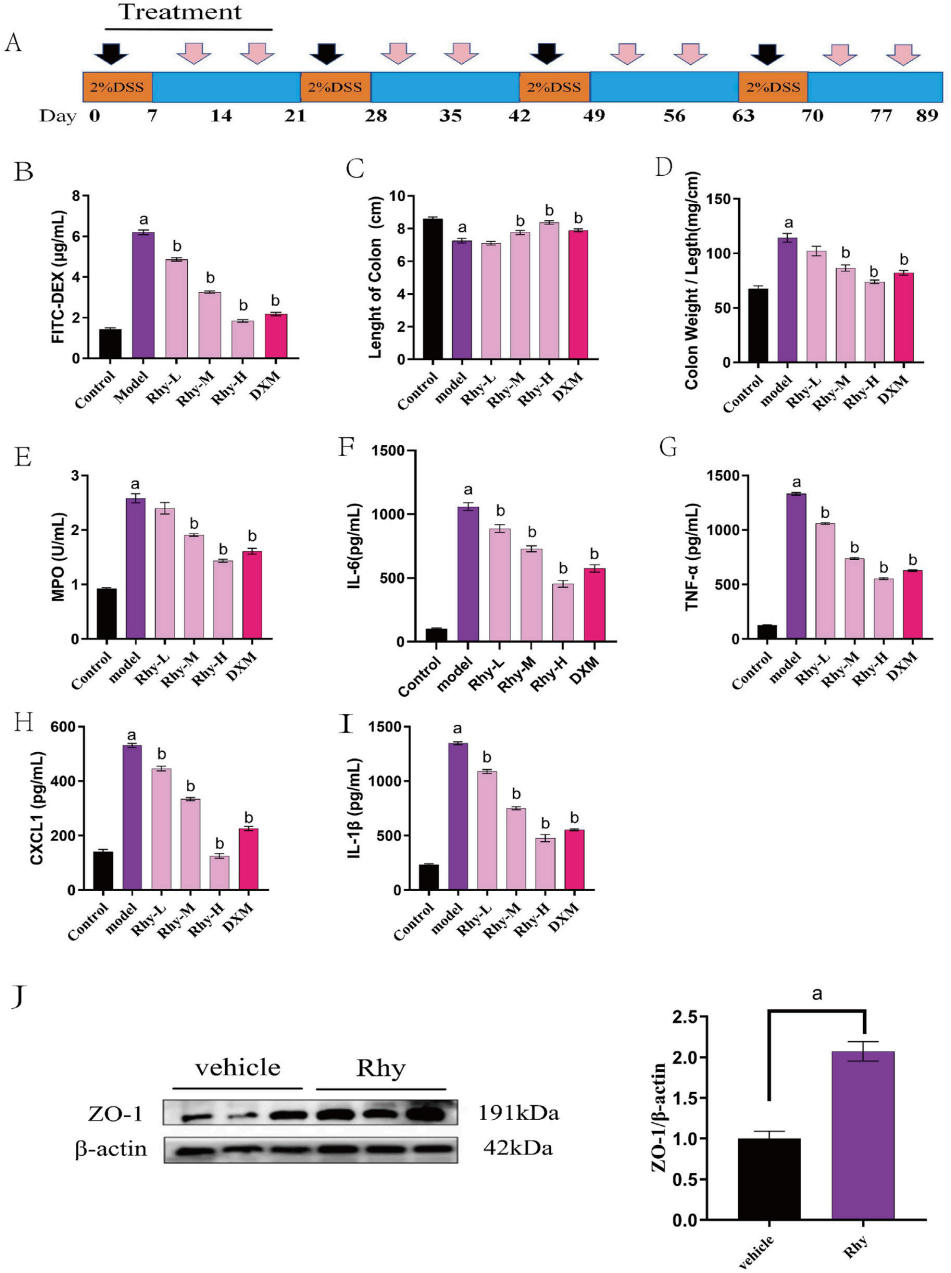
Rhy prevent DSS-induced chronic colitis. **(A)** Methods of chronic colitis modeling and drug administration (n = 10). **(B)** Intestinal permeability was evaluated using FITC-dextran leakage assay. **(C)** Colon lengths. **(D)** Ratio of colon weight/length. **(E)** Effect of Rhy on the levels of inflammatory factors MPO in the colon homogenate of IBD mice. **(F)** IL-6 level. **(G)** TNF-α level. **(H)** CXCL-1 level. **(I)** IL-1β level. **(J)** ZO-1 expression in the colons of these mice was measured by immunoblots and quantified (n = 3). ^a^ *P* < 0.05, indicating comparison with control group, ^b^ *P* < 0.05, indicating comparison with model group. Data were expressed as mean ± SEM.

The therapeutic effect of Rhy was also examined in a chronic colitis model induced by 2% DSS. Mice consumed 2% DSS water for four cycles, each lasting 7 days with a 14-day interval of normal water consumption between cycles (Figure 3A). Rhy treatment significantly protected against chronic colitis in mice, reducing intestinal permeability, colonic shortening, and the colon weight/length ratio (Figures 3B–D). It also lowered serum levels of inflammatory cytokines IL-6, TNF-α, CXCL1, IL-1β (Figures 3F–I) and colonic tissue MPO levels (Figure 3E). Rhy was shown to upregulate the expression of the intestinal barrier protein ZO-1 (Figure 3J). The results suggest that Rhy enhances intestinal barrier integrity and alleviates symptoms of chronic colitis.

### 3.4 Rhy prevents colitis in mice

**FIGURE 4:**
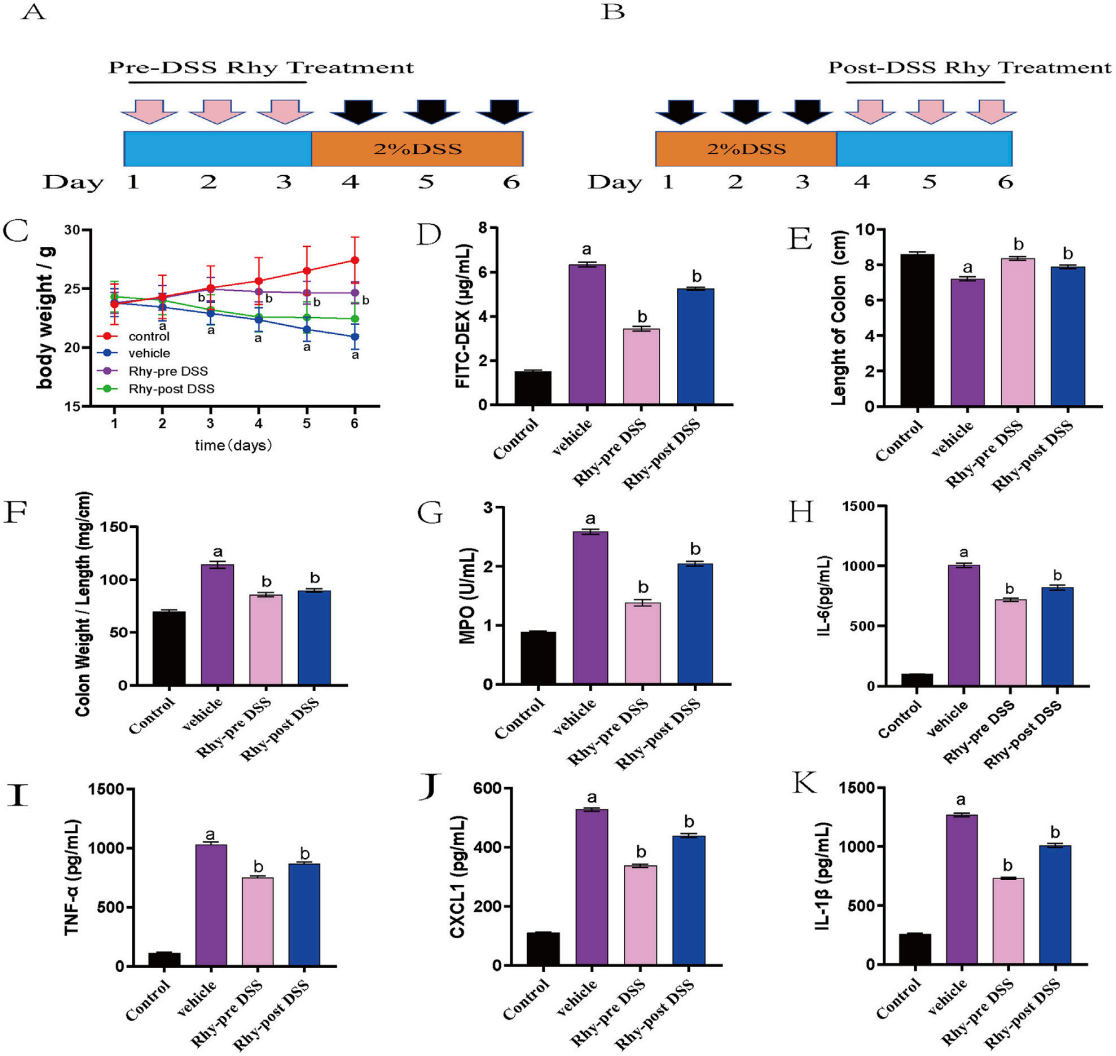
Rhy prevent DSS-induced colitis and sustain beneficial barrier activities (n = 10). **(A)** Pre-DSS treatment. **(B)** Post-DSS treatment. **(C)** Percent body weight loss was recorded after DSS-administration. **(D)** Intestinal permeability was evaluated using FITC-dextran leakage assay. **(E)** Colon lengths. **(F)** Ratio of colon weight/length. **(G)** Effect of Rhy on the levels of inflammatory factors MPO in the colon homogenate of IBD mice. **(H)** IL-6 level. **(I)** TNF-α level. **(J)** CXCL-1 level. **(K)** IL-1β level. ^a^ *P* < 0.05, indicating comparison with control group, ^b^ *P* < 0.05, indicating comparison with model group. Data were expressed as mean ± SEM.

Due to the strong intestinal barrier protective activity demonstrated Rhy through the upregulation of tight junction proteins, we investigated the preventative effects of Rhy in a DSS-induced colitis model. Mice were pre-treated orally with Rhy for 3 days prior to a 3-day DSS induction of colitis, during which no further Rhy treatment was administered (Figure 4A). Another treatment regimen involved inducing colitis with DSS for 3 days, followed by a 3-day oral administration of Rhy (Figure 4B). Results from both treatment regimens showed that Rhy significantly protected mice from DSS-induced weight loss, decreased intestinal permeability, colonic shortening, and an increase in the colon length/weight ratio (Figures 4C–F), and it also reduced colonic inflammatory cytokine levels (Figures 4G–K). However, the study found that prophylactic administration of Rhy was more effective than therapeutic administration.

### 3.5 Rhy enhances intestinal barrier function via AhR mediation

**FIGURE 5:**
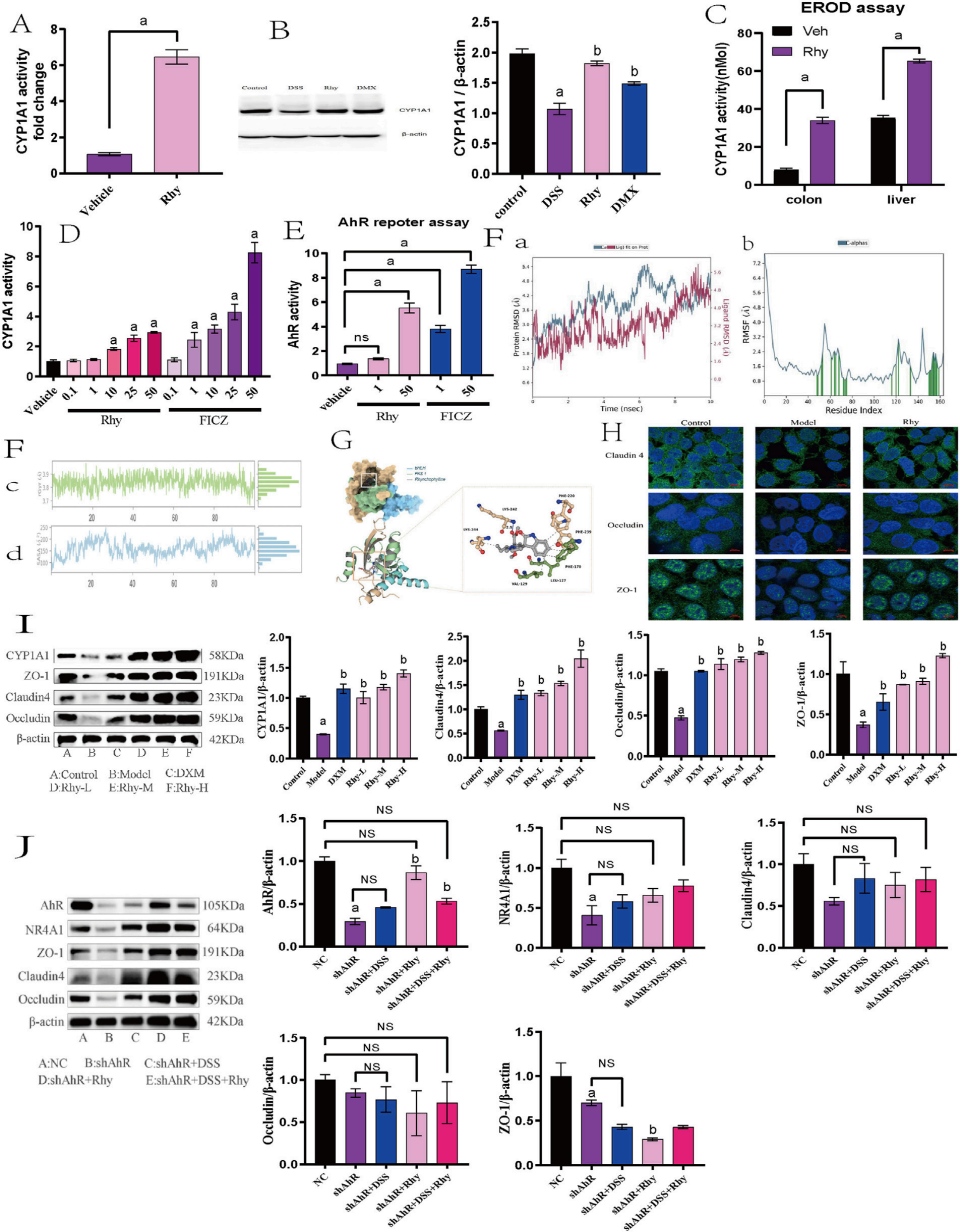
Rhy enhance tight junction proteins in AhR-dependent manner. **(A)** mRNA levels of Cytochrome P450 1A1 (Cyp1A1) was measured by RT-PCR (n = 7). **(B)** Cyp1A1 protein levels were measured using immunoblots and quantified (n = 3). **(C)** Cyp1A1 activity was measured in colons and livers by ethoxyresorufin-O-deethylase (EROD) assay (n = 6). **(D)** Cyp1A1 enzyme activity was measured by P450-Glo Cyp1A1 assay (n = 3). **(E)** The cells expressing AhR-reporter (luciferase) were treated with Rhy or FICZ (AhR high affinity ligand) and fold change of luminescence over vehicle treatment was measured (n = 3). **(F)** Molecular dynamics simulation of Rhy and AhR. **(A)**. RMSD system of Rhy-AhR; **(B)**. RMSF system of Rhy-AhR; **(C)**. Rhy-AhR system radius of rotation; **(D)**. Rhy-AhR system solvent can reach surface area. **(G)** Interaction between Rhy and AHR. **(H)** Effect of Rhy on intestinal barrier related proteins Claudin4, Occludin and ZO-1 (n = 3). **(I)** Effects of Rhy on Cyp1A1 and intestinal barrier related proteins (n = 3). **(J)** Expression of AhR, NR4A1protein and intestinal barrier protein after knocking down AhR (n = 3). ^a^ *P* < 0.05, indicating comparison with control group, ^b^ *P* < 0.05, indicating comparison with model group. Data were expressed as mean ± SEM.

Given that CYP1A1 is a key downstream effector of the AhR signaling pathway, the analysis of CYP1A1 activity provides insights into whether Rhy activates AhR to enhance intestinal barrier function. Real-Time PCR data indicated a significant increase in CYP1A1 mRNA levels in response to Rhy treatment (Figure 5A). Western blot results also showed a substantial upregulation of CYP1A1 protein in colonic epithelial cells following Rhy administration (Figure 5B). In the P450-Glo assay, the effect of Rhy on CYP1A1 activity was compared using 6-formylindolo [3,2-b]carbazole (FICZ), a strong AhR activator. The findings revealed that Rhy significantly enhanced CYP1A1 activity in colonic epithelial cells, albeit less effectively than FICZ. Through EROD assays, the impact of Rhy on CYP1A1 activity *in vivo* was assessed, showing notable increases in CYP1A1 activity in both the colon and liver of mice treated with Rhy(Figure 5C). FICZ activated CYP1A1 approximately three times more than Rhy did in the colon (Figure 5D).

These observations confirm that Rhy not only boosts CYP1A1 expression but also enhances its enzymatic activity through AhR activation. Additionally, in the Caco-2 cell line, the direct activation of AhR by Rhy was evaluated using an XRE-luciferase reporter gene system. Data indicated that 50 µM Rhy triggered a five-fold increase in luciferase activity compared to the vehicle, demonstrating significant AhR activation. In contrast, 50 µM FICZ led to a nine-fold increase in luciferase activity, highlighting a higher level of AhR activation by the potent agonist FICZ compared to Rhy treatment (Figure 5E).

Molecular dynamics simulations of the Rhy-AhR system demonstrated structural stability and overall kinetic stability, which is conducive to the effective pharmacological action of Rhy (Figure 5F). Molecular docking of Rhy with AhR primarily involves the bHLH (27–80) and PAS 1 (111–181) domains of the protein’s A-chain, represented in blue and green, respectively, in the figure (Figure 5G). The docking score for Rhy with AhR was −8.8 kcal/mol, and the binding free energy was −35.25 kcal/mol. Besides hydrogen bonding, hydrophobic interactions between hydrophobic amino acids and the ligand groups predominantly influence the protein-ligand binding. A hydrogen bond is formed between the carboxyl oxygen atom of Rhy and LYS-242 of AhR, along with hydrophobic interactions with LYS-244, VAL-129, LEU-127, PHE-170, PHE-239, and PHE-220.

Immunofluorescence experiments revealed that the proteins Claudin4, Occludin, and ZO-1 are primarily located at the intercellular contact areas, forming continuous linear structures that regulate paracellular permeability. The cell nuclei appeared as blue dots stained with DAPI. Compared to the control group, the fluorescence intensity of Claudin4, Occludin, and ZO-1 was reduced in the model group; following Rhy intervention, the fluorescence intensity of Claudin4, Occludin, and ZO-1 increased compared to the model group (Figure 5H).

Western blot results revealed that in a DSS-induced acute colitis mouse model, compared to normal controls, the expression levels of Cyp1A1 and intestinal barrier proteins such as ZO-1, Claudin4, and Occludin were significantly decreased. Treatment with Rhy notably enhanced the expression of these proteins in a dose-dependent manner (Figure 5I). To investigate whether Rhy’s upregulation of the tight junction proteins ZO-1, Claudin4, and Occludin is dependent on AhR activation, siRNA technology was employed to suppress AhR expression, and the levels of these proteins were subsequently measured. Experimental data revealed that in cells treated with siRNA targeting AhR, Rhy failed to effectively enhance the expression of ZO-1, Claudin4, and Occludin, suggesting that AhR activation is a critical mediator of Rhy’s effects (Figure 5J). This indicates that Rhy’s beneficial impact on intestinal barrier integrity is mediated through its activation of the AhR pathway.

### 3.6 Rhy enhances intestinal barrier function through NR4A1 mediation

**FIGURE 6:**
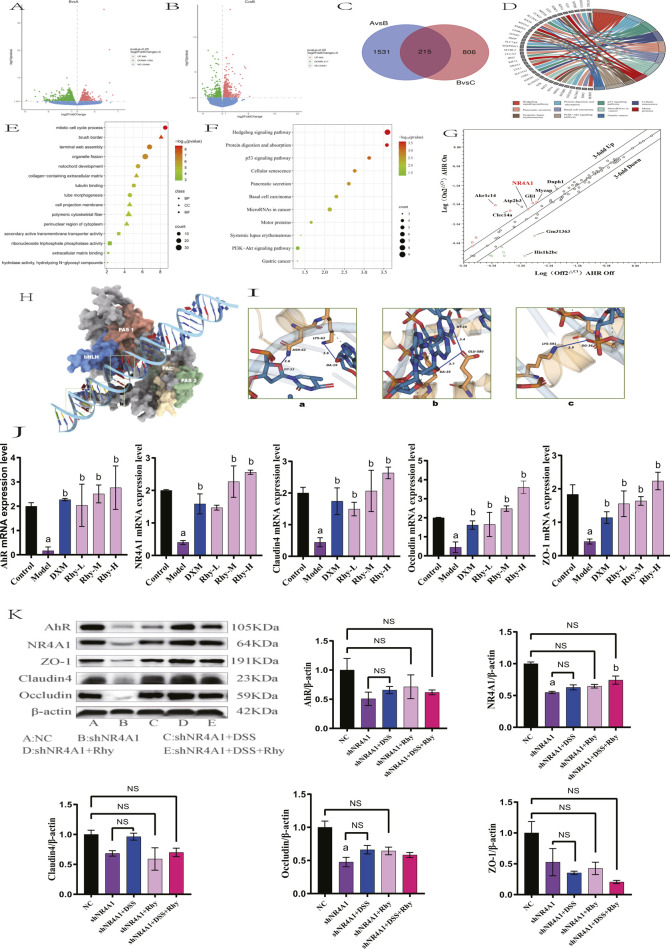
NR4A1 is required for Rhy mediated upregulation of tight junction proteins. **(A)** Differential gene expression between control group and model group (n = 3). **(B)** Differential gene expression between model group and Rhy group (n = 3). A is control group, B is model group, C is Rhy group. **(C)** The intersection of different genes in control group, model group and Rhy group. **(D)** Differential genes are expressed in the pathway. **(E)** GO Analysis of differential genes in colon tissue of IBD mice. **(F)** KEGG Analysis of differential genes in colon tissue of IBD mice. **(G)** A focused RT-QPCR screen of gene expression in the AhR-inducible (n = 3). **(H)** Three-dimensional structure of AhR and NR4A1. **(I)** AhR interacts with the NR4A1 complex. **(J)** Effect of Rhynchophylline on NR4A1 and intestinal barrier related mRNA in NCM460 cells (n = 3). **(K)** Expression of AhR, NR4A1protein and intestinal barrier protein after knocking down NR4A1 (n = 3). ^a^ *P* < 0.05, indicating comparison with control group, ^b^ *P* < 0.05, indicating comparison with model group. Data were expressed as mean ± SEM.

Further RNA-Seq analysis of colonic tissues from IBD mice revealed that between the control group and the model group, there were 1746 differentially expressed mRNAs, with 1056 genes upregulated and 690 downregulated (Figure 6A). Between the Rhy group and the model group, there were 1021 differentially expressed mRNAs, with 417 upregulated and 604 downregulated (Figure 6B). An intersection of these revealed 215 differentially expressed mRNAs (Figure 6C). Further enrichment analysis of these differentially expressed mRNAs through GO and KEGG pathways showed involvement in processes like extracellular matrix binding, aggregation of cytoskeletal filaments, and the mitotic cell cycle (Figure 6D). KEGG enrichment indicated that the intersected targets are involved in classic signaling pathways such as PI3K-Akt and P53, as well as novel pathways like Hedgehog and MicroRNAs in cancer (Figures 6E,F). Among them, five differentially expressed mRNAs: NR4A1, Lpar1, Ccnd2, Fgfr2, and Epha2 were enriched in the PI3K-Akt signaling pathway, with NR4A1 expression increased by 2.5-fold (Figure 6G). Based on these results, we hypothesize that NR4A1 is a downstream target of AhR, and Rhy enhances intestinal barrier function through the AhR-NR4A1 pathway. This hypothesis was validated in colonic epithelial cells with knocked-down AhR, where NR4A1 expression decreased and Rhy treatment did not induce an upregulation of NR4A1 protein expression (Figure 5J).

Since AhR is essential for Rhy-mediated activity, we hypothesized that AhR binds to the NR4A1 promoter sequence. Based on predictions from the MEME Suite, we successfully identified the binding sites between AhR and NR4A1, including the binding site of AhR_HUMAN (612AA-660AA) and the NR4A1 promoter sequence (TTT​TAT​TAT​TTA​TTT​ATA​ATT​TAT​TTT​TAT​TTT​TAG​AGA​TGT​GTC​TTG​C). Through modeling and protein-DNA docking simulations, we obtained the three-dimensional structure of the AhR-NR4A1 complex. The docking score and confidence score of AhR-NR4A1 were −219.22 and 0.7997, respectively, suggesting a high probability of binding between the two molecules (Figure 6H). Systematic analysis of the binding interface of the complex using the PLIP tool showed that the interface primarily consists of two types of interactions: hydrogen bonds (blue solid lines) between LYS-63 of AhR and DA-19 of NR4A1, and ASN-62 of AhR and DT-33 of NR4A1; GLU-580 of AhR forms hydrogen bonds with DT-34 and DA-35 of NR4A1; and LYS-591 of AhR forms a hydrogen bond with DG-36 of NR4A1, in addition to multiple hydrophobic interactions (grey dashed lines) (Figure 6I).

qPCR findings demonstrated that, relative to the control group, there was a significant reduction in NR4A1 mRNA expression and intestinal barrier protein mRNA levels, including ZO-1, Claudin4, and Occludin, in the model group. Treatment with Rhy significantly upregulated NR4A1 mRNA expression and the mRNA levels of intestinal barrier proteins ZO-1, Claudin4, and Occludin in a dose-dependent manner (Figure 6J). In contrast, Rhy was unable to induce upregulation of these intestinal barrier proteins ZO-1, Claudin4, Occludin in NR4A1-knockdown cells (Figure 6K). Overall, these results indicate that the upregulation of tight junction proteins mediated by Rhy is dependent on AhR and NR4A1. Therefore, Rhy enhances intestinal barrier function and alleviates inflammation through the AhR-NR4A1 pathway, playing a therapeutic role in the treatment of IBD.

## 4 Discussion

In this study, we demonstrated that Rhynchophylline (Rhy) not only exhibits anti-inflammatory effects but also promotes intestinal health by enhancing barrier function. Our findings further confirm that Rhy activates the AhR-NR4A1 signaling pathway, which enhances the expression of tight junction proteins and suppresses inflammatory responses. Additionally, Rhy has shown significant efficacy in both the prevention and treatment of colitis. Rhy, a bioactive alkaloid extracted from the traditional Chinese herb Uncaria rhynchophylla, possesses pharmacological activities such as calcium channel blocking, anti-inflammatory effects, antihypertensive properties, and ischemic protection (Kaneko et al., 2020; Zhou and Zhou, 2010; Wastag et al., 2022). However, the molecular targets of Rhy in the pathophysiology of IBD have yet to be fully explained. Through RNA-Seq analysis, we uncovered the protective effects of Rhy on colonic epithelial cells and the potential mechanisms involved.

Rhy mediates the upregulation of tight junction proteins such as ZO-1, Claudin4, and Occludin, providing protection against the “leaky gut” phenotype induced by LPS in colonic epithelia, thus playing a crucial role in regulating intestinal barrier function. The intestinal barrier comprises colonic epithelial cells and the connections between these cells, including gap junctions, adherens junctions, and tight junctions, which collectively function to seal intercellular spaces (Li et al., 2022; Vindigni et al., 2016). Tight junctions consist of proteins such as Occludin, Claudins, and Zona Occludens-1 (ZO-1) (Chen et al., 2021; Peng et al., 2023). When the function of the intestinal barrier is compromised, it can lead to dysbiosis of the gut microbiota, allowing various harmful substances to cross the intestinal barrier into the bloodstream, causing the invasion of pathogens, endotoxins, and allergens into the intestinal lumen. This results in excessive immune responses and inflammatory reactions (Camilleri et al., 2012; Mehandru and Colombel, 2021). In patients with Crohn’s disease, proteins such as Occludin, Claudin-3, -5, -8 are notably decreased and redistributed (Kuo et al., 2019). Similarly, in patients with ulcerative colitis, expressions of Occludin, Claudin-1, and -4 are found to be reduced (Wei et al., 2021). Dysfunction of the barrier function and inflammation create a vicious cycle in IBD, and blocking one pathway is often insufficient to alleviate the disease progression (Di Vincenzo et al., 2024). Therefore, repairing the integrity of the intestinal tight junctions as a therapeutic strategy for IBD may be effective.

RNA-seq analysis revealed that Rhy not only enhances the expression of tight junction proteins ZO-1, Claudin4, and Occludin in colonic epithelial cells but also upregulates the expression of Cyp1A1. Given that Cyp1A1 is a liver enzyme involved in Phase I drug metabolism and a downstream gene of AhR (AHR, 2024), these findings imply that AhR might mediate the effects of Rhy. Upon activation, AhR translocates to the nucleus, where it forms heterodimers with the AhR Nuclear Translocator (ARNT). This complex specifically binds to AhR response elements, thereby initiating the transcription of various target genes, including CYP1A1 (Sondermann et al., 2023; Alhamad et al., 2022). Research by Singh et al. (Singh et al., 2011) demonstrated that activating AhR can inhibit the MLCK-pMLC signaling pathway, which prevents the disruption of tight junctions caused by TNF-α and IFN-γ. This activation promotes increased expression of ZO-1, Occludin, and Claudin proteins, reduces intestinal mucosal permeability, and helps repair the intestinal barrier. Furthermore, activated AhR enhances the expression of IL-22, which helps suppress intestinal inflammation in mice with colitis (Lee et al., 2011). Activation of AhR by Tetrachlorodibenzo-p-dioxin (TCDD) has been shown to alleviate intestinal inflammation in Crohn’s disease (Swigonska et al., 2023). The AhR agonist β-naphthoflavone significantly reduces colitis induced by DSS (El-Dairi et al., 2020). Treatment with FICZ not only alleviates DSS-induced colitis but also reduces colitis induced by 2,4,6-trinitrobenzenesulfonic acid (TNBS) (MiR-124a Mediates the Impairment, 2024). Conversely, administering an AhR antagonist to mice worsens TNBS-induced colitis (Benson and Shepherd, 2011), suggesting that AhR activation can mitigate intestinal inflammation, thus proposing AhR as a ‘new target’ for IBD treatment. Studies show that Rhy induces the expression and nuclear translocation of AhR, enhances the transcription of XRE target genes, and induces Cyp1A1 enzyme activity.

What is Interesting is that Rhy failed to induce the expression of ZO-1, Occludin, and Claudin4 proteins in AhR-knockdown cells. Previous reports have indicated that overexpression of Cyp1A1 in mice leads to a reduction in AhR ligands, resulting in decreased AhR activity, a form of negative feedback regulation (Stockinger et al., 2021). Although Cyp1A1 activity increased following Rhy treatment, we did not observe a decrease in Rhy’s pharmacological activity consequent to increased Cyp1A1 activity. It is also possible that Rhy is metabolized through Cyp1A1, producing unknown active metabolites that activate the AhR-NR4A1 pathway. Wei Wang and others have reported (Wang et al., 2010) that 24 h after oral administration, 9.4% of Rhy is metabolized into 11-hydroxyRhynchophylline and 10-hydroxyRhynchophylline in roughly equal proportions. Cytochrome P450 (CYP) enzymes in rat liver microsomes play a crucial role in the hydroxylation of Rhy, with CYP2D, CYP1A1/2, and CYP2C involved in the hydroxylation process (Liu et al., 2023). The metabolites of Rhy might also exhibit certain beneficial activities. Further research is required to support these possibilities, including the specific knockout of CYP1 enzymes in systemic and intestinal epithelial cells to determine the exact role of Cyp1A1 in Rhy-mediated activities.

Rhy is unable to exert its activity in cells with knocked-down AhR, indicating that the AhR pathway plays a crucial role in mediating Rhy’s effects. While previous studies have confirmed the role of AhR in regulating immune cell functions (The aryl hydrocarbon receptor, 2024; Gao et al., 2018), our research highlights the importance of AhR in regulating tight junction proteins and intestinal barrier functions in colonic epithelial cells. NR4A1, as an anti-inflammatory gene, are vital in the body’s immune response (Pulakazhi Venu et al., 2021; Guo et al., 2021). The expression levels of the NR4A1 gene are significantly reduced in various cancer tissues such as bladder urothelial carcinoma, invasive breast cancer, and head and neck squamous cell carcinoma (Deng et al., 2022). NR4A1 is closely associated with inflammatory processes and exerts a significant inhibitory effect in monocytes through the nuclear factor kappa-B (NF-κB) signaling pathway (Liu et al., 2019). Genetic variations in NR4A1 are associated with IBD risk, and activation of NR4A1 has been proven to regulate intestinal inflammation (Wu et al., 2016). Current research focuses on identifying AhR’s target genes and their associated regulatory factors. Whether NR4A1 acts as a downstream effector gene of AhR is a focal point of this study. Previous studies have shown that the complex formed by AhR and ARNT can bind to the promoter region of NR4A1, thereby increasing its transcriptional expression (Safe et al., 2023). Our *in vitro* and *in vivo* results indicate that Rhy significantly induces the expression of AhR and its target gene NR4A1 in colonic epithelial cells. Moreover, the AhR-NR4A1 pathway is crucial for the upregulation of tight junction proteins mediated by Rhy.

Animal studies have demonstrated that Rhy treatment significantly enhances tight junction proteins, reduces intestinal permeability, and diminishes local and systemic inflammation, thus alleviating symptoms of colitis. A dosage of 2 mg kg^-1^ Rhy has shown therapeutic effects on DSS-induced colitis, and it has been observed that Rhy enhances intestinal barrier function, which helps prevent the development of colitis. In mice pre-treated with Rhy before DSS-induced colitis, there was a significant reduction in intestinal permeability, consistent with Rhy’s increase in the expression of tight junction proteins. Even after discontinuing Rhy treatment post-DSS-induced colitis, mice still showed delayed progression of the disease, suggesting that Rhy may exert preventive effects on colitis by enhancing intestinal barrier function. Daily administration of Rhy resulted in upregulated expression of AhR, NR4A1, and tight junction proteins such as ZO-1, Occludin, and Claudin4 in the colons of mice. These findings highlight Rhy’s potential to strengthen intestinal barrier integrity, which could play a crucial role in preventing the onset of colitis. Rhy not only mitigates both chronic and acute colitis induced by DSS but also demonstrates efficacy across different models, indicating its broad benefits. Unlike high-affinity and potentially toxic AhR ligands like FICZ, Rhy, as a low-affinity and non-toxic AhR ligand, offers a novel therapeutic strategy for treating colitis.

Rhy was unable to upregulate the expression of DSS-induced intestinal barrier proteins ZO-1, Occludin, and Claudin4 in cells with knocked-down AhR or NR4A1, indicating that the AhR-NR4A1 pathway mediates Rhy’s activities in repairing the intestinal barrier and its anti-inflammatory effects. Rhy may exert its anti-colitis effects through two mechanisms. Firstly, Rhy acts directly on immune cells, such as macrophages, to prevent inflammation induced by LPS or bacteria, and exhibits anti-inflammatory activity through the AhR-NR4A1 pathway. Most importantly, Rhy directly affects intestinal epithelium and barrier function by upregulating tight junction proteins. Enhanced barrier function can reduce bacterial leakage within the intestine, thereby significantly reducing systemic inflammation. RNA-Seq pathway analysis indicates that Rhy could also be crucial in inducing the Hedgehog signaling pathway. Recent studies have demonstrated a close connection between the Hedgehog signaling pathway and the pathogenesis of inflammatory bowel disease (IBD) (Buongusto et al., 2017; Eriodictyol attenuates dextran sodium sulphate, 2024). The activation of this pathway significantly influences the proliferation and differentiation of intestinal stem cells, which are crucial for the repair and regeneration of the intestinal epithelium (Bouwman et al., 2022). Moreover, the Hedgehog signaling pathway may also be involved in the development of IBD by regulating immune responses and inflammatory processes (Lees et al., 2008). Thus, we hypothesize that besides its anti-inflammatory and barrier-protective properties, Rhy could treat IBD by modulating the Hedgehog signaling pathway.

## 5 Conclusion

This research summarizes how Rhy alleviates IBD by improving the function of intestinal barrier and reducing inflammation. Current IBD treatments focus on suppressing inflammation, but enhancing intestinal barrier function might be a more effective therapeutic approach. In conclusion, Rhy has shown positive effects in treating inflammatory bowel disease (IBD) and its potential clinical applications may extend beyond this condition. Rhy could also be beneficial for other diseases caused by barrier dysfunction and inflammation, such as alcoholic liver disease, neurological disorders, and colon cancer. These findings underscore Rhy’s potential therapeutic role across multiple disease contexts, highlighting its significant translational relevance.

## Data Availability

The original contributions presented in the study are publicly available. This data can be found here: Gene Expression Omnibus (GEO), accession number GSE307290.
